# ФРФ23-индуцированная остеомаляция опухолевого генеза

**DOI:** 10.14341/probl13130

**Published:** 2022-07-11

**Authors:** С. А. Гронская, Ж. Е. Белая, Г. А. Мельниченко

**Affiliations:** Научный медицинский исследовательский центр эндокринологии; Научный медицинский исследовательский центр эндокринологии; Научный медицинский исследовательский центр эндокринологии

**Keywords:** фосфопения, фосфор, остеомаляция, опухоль, ФРФ23, гиперпаратиреоз, остеопороз

## Abstract

Фосфопеническая остеомаляция опухолевого генеза — редкое приобретенное заболевание. Причиной является мезенхимальная опухоль, секретирующая фактор роста фибробластов 23 (ФРФ23). Избыточное количество ФРФ23 нарушает метаболизм фосфора и витамина D, что приводит к тяжелому паранеопластическому синдрому, проявляющемуся в виде множественных переломов, выраженного болевого синдрома в костях и генерализованной миопатии. Возможно полное излечение при радикальной резекции опухоли. К сожалению, локализация, малый размер образований и редкая встречаемость приводят к тому, что заболевание длительно остается нераспознанным и приводит к тяжелым, инвалидизирующим последствиям. Поэтапный подход к диагностике улучшает результаты. Вначале производят тщательный сбор анамнеза и лабораторную диагностику, затем функциональную визуализацию и подтверждают диагноз анатомической визуализацией опухоли. Методом выбора является оперативное лечение. Если резекция невозможна, то показана медикаментозная терапия активными метаболитами витамина D и солями фосфора. Активно развиваются новые терапевтические и диагностические подходы, такие как антитело к ФРФ23 и пан-ингибиторы рецепторов ФРФ. В данной статье представлен обзор современных подходов к диагностике и лечению ФРФ23-индуцированной остеомаляции.

## Abstract (en)

Tumor induced osteomalacia is a rare acquired disease. The cause is a mesenchymal tumor secreting fibroblast growth factor 23 (FGF23). An excessive amount of FGF 23 disrupts the metabolism of phosphorus and vitamin D, which leads to severe paraneoplastic syndrome, manifested in the form of multiple fractures, severe pain in the bones and generalized myopathy. With oncogenic osteomalacia, a complete cure is possible with radical resection of the tumor. Unfortunately, localization, small size of formations and rare frequency of occurrence lead to the fact that the disease remains unrecognized for a long time and leads to severe, disabling consequences. A step-by-step approach to diagnosis improves treatment outcomes. First, a thorough anamnesis is collected, then functional visualization is performed and the diagnosis is confirmed by anatomical visualization of the tumor. After that, the method of choice is a surgical treatment. If resection is not possible, then conservative therapy with active metabolites of vitamin D and phosphorus salts is indicated. New therapeutic approaches, such as the antibody to FGF23 or the pan-inhibitor of receptors to FGF, are actively developing. This article provides an overview of modern approaches to the diagnosis and treatment of this disease.

## ВВЕДЕНИЕ

Фосфопеническая остеомаляция опухолевого генеза относится к орфанным, метаболическим заболеваниям [1][2]. На фоне избыточной секреции фактора роста фибробластов 23 (ФРФ23, fibroblast growth factor 23, FGF23) развивается тяжелый паранеопластический синдром, который в литературе называют «опухоль-индуцированной остеомаляцией» или «онкогенной остеомаляцией» (Tumor induced osteomalacia, TIO). Причиной является мезенхимальная опухоль, секретирующая ФРФ23. В большинстве случаев это высокодифференцированные, доброкачественные новообразования с умеренным темпом роста. Однако, несмотря на доброкачественность гистоморфологической картины, заболевание протекает тяжело как за счет гиперсекреции ФРФ23, так и за счет возможного метастазирования (вероятно, в случае повреждения целостности образования). Гиперсекреция ФРФ23 приводит к дефициту ионов фосфата и, соответственно, недостаточной минерализации костей, т.е.к остеомаляции [3–7].

Опухолевая остеомаляция встречается, как правило, во взрослом возрасте и требует тщательной дифференциальной диагностики, которая будет подробно освещена ниже. Впервые заболевание было описано достаточно поздно, в конце XX в. (схожий по клинике рахит описан в XVII в. [8]). У пациентов наблюдаются выраженные боли в костях (до 99,3%), множественные переломы (до 79%), уменьшение в росте (до 69%), генерализованная миопатия (до 65%), а также ряд неспецифических симптомов [1][3][4][9–11]. Визуализация опухолей с помощью мультиспиральной компьютерной томографии (МСКТ) или магнитно-резонансной томографии (МРТ) сложна из-за малых размеров, мезенхимальной природы образований и разнообразной локализации, как внутри костей так и в мягких тканях. Вдобавок неспецифичность клинической картины и малая распространенность приводят к тому, что заболевание остается нераспознанным [10–12]. Активная диагностика началась с 2008 г., что связано с прогрессом инструментальных методов. В мире известно около 1725 клинических случаев [13][14]. Распространенность и эпидемиология из-за редкости изучены недостаточно и неизвестны.

Полное излечение возможно при радикальном удалении опухоли, в отличие от генетически обусловленных форм остеомаляции [15][16]. Отсутствие ремиссии наблюдается при невозможности удалить патологический очаг, а рецидивы чаще встречаются при нерадикальном хирургическом лечении и метастазировании. В таких случаях показана медикаментозная терапия [2].

Описаны случаи, когда ФРФ23 синтезировался злокачественными образованиями (рак простаты [17], легкого [18], яичников [19], гемобластоз [20] и др.). Есть данные, что встречаются «ФРФ23-негативные» опухоли, секретирующие другие фосфатрегулирующие соединения: frizzled-related protein 4, FGF7 и матриксный внеклеточный фосфогликопротеин [21–23].

Подробная информация о ФРФ23-секретирующих опухолях представлена в данном обзоре.

## ФАКТОРЫ РОСТА ФИБРОБЛАСТОВ. МЕТАБОЛИЗМ И РОЛЬ ФРФ23

ФРФ23 — белок из семейства факторов роста фибробластов (ФРФ), в котором известно 23 члена. Первый представитель — ФРФ2 — был открыт в 1973 г. и широко используется в микробиологии по сей день [24][25]. Большинство ФРФ действуют паракринно, однако некоторые (ФРФ23, ФРФ21, ФРФ19) обладают эндокринными свойствами [26]. Члены семейства имеют структурное сходство и участвуют в ангиогенезе, эмбриогенезе, регенерации и метаболизме, взаимодействуя с рецепторами FGFR1, FGFR2, FGFR3, и FGFR4 [26][27]. Разнообразие биологических функций ФРФ обусловлено различиями рецепторов и кофакторов, образующих множество комбинаций и активизирующих специфические сигнальные пути [28–30]. ФРФ являются ценными мишенями для разработки лекарственных веществ. Уже сейчас доступны препараты для лечения ожоговых и язвенных ран, разрабатываются регенерирующие и противоопухолевые средства на основе ФРФ [26][31].

В данном обзоре мы более подробно рассмотрим ФРФ23. Названия белков будут написаны заглавными буквами (например, FGF23). Названия генов будут написаны заглавными курсивными буквами (например, FGF23). ФРФ23 открыт в 2000 г. японским исследователем T. Yamashita [32]. ФРФ23 был обнаружен в мышином вентролатеральном ядре таламуса и привлек к себе внимание ученых. После ряда экспериментов ФРФ23 идентифицировали как причину аутосомно-доминантного рахита [33], oпухоль-индуцированной остеомаляции [34], показали его ведущую роль в патогенезе почечной недостаточности [5]. Клетки костной ткани (остеоциты и остеобласты) синтезируют белок ФРФ23. Концентрация ФРФ23 регулируется на множестве биологических уровней (в процессе транскрипции, посттрансляционно, в системном кровотоке), что свидетельствует о его важности для организма. На процесс транскрипции ФРФ23 оказывают влияние белки DMP-1, ANKH, PHEX, ENPP1, а мутации вих генах приводят к заболеваниям (табл. 1) [35–42]. В результате транскрипции гена FGF23, расположенного на 12-й хромосоме и состоящего из 3 экзонов, синтезируется белок, состоящий из 251 аминокислоты (32 kD) [26]. Далее включаются механизмы посттрансляционной модификации: отщепляется сигнальный пептид (24 амк) и ФРФ23 становится биоактивным. Часть ФРФ23 инактивируется внутри клетки, а часть секретируется в кровь в биоактивной форме (рис. 1). Инактивация внутри клетки происходит путем фосфорилирования белком Fam20C (FAM20C), а затем расщепления белком FURIN-like proteasa на N-терминальный (154 амк) и С-терминальный (73 амк) пептиды [43]. Баланс между биоактивной и инактивированной формами ФРФ23 крайне важен и предположительно зависит от уровня фосфора крови. Высокие концентрации фосфора опосредованно повышают концентрацию ФРФ23, увеличивая экспрессию белка GalNAc-N3 (GALNT3). GalNAc-N3 О-гликозилирует биоактивный ФРФ23, делая его недоступным для фосфорилирования и протеолиза, тем самым повышая концентрацию биоактивной формы в крови (рис. 1) [44].

**Table:** Таблица 1. Заболевания, обусловленные ФРФ23Table 1. Diseases due to FGF23 Примечание. АР — аутосомно-рецессивный тип наследования; АД — аутосомно-доминантный тип наследования.

Заболевание	Аббревиатура	Тип	Ген	Механизм	Комментарии	OMIM
Дефицит ФРФ23 и гиперфосфатемия
Первичный опухолевый (туморальный) кальциноз тип 1	HFTC, HSS	АР	GALNT3	Дефицит ФРФ23	Отсутствует О-гликозилирование ФРФ23, в итоге избыточная инактивация ФРФ23	#211900 #610233
Первичный опухолевый (туморальный) кальциноз тип 2	HFTC	АР	FGF23	Дефицит ФРФ23	Мутации ФРФ23	#211900
Первичный опухолевый (туморальный) кальциноз тип 3	HFTC	АР транслокация	KL	Дефицит ФРФ23	Мутации Klotho	#211900
Избыток ФРФ23 и гипофосфатемия
Х-связанный гипофосфатемический рахит	XLH	X-сцепленно	PHEX	Избыток ФРФ23	Мутации в PHEX стимулируют избыточную транскрипцию ФРФ23	#307800
Аутосомно-доминантный гипофосфатемический рахит	ADHR	АД	FGF23	Избыток ФРФ23	Мутантный белок ФРФ23 резистентен к инактивации и протеолизу	#193100
Гипофосфатемический рахит с гиперпаратиреозом	ADHR	АД	KL	Избыток ФРФ23	Избыток ФРФ23	#612089
Аутосомно-рецессивный гипофосфатемический рахит 1 типа	ARHP	АР	DMP1	Избыток ФРФ23	Мутации в генах стимулируют избыточную транскрипцию ФРФ23	#241520
Аутосомно-рецессивный гипофосфатемический рахит 2 типа	ARHP	АР	ENPP1	Избыток ФРФ23	Мутации в генах стимулируют избыточную транскрипцию ФРФ23	#173335
Болезнь депонирования кристаллов пирофосфата кальция; хондрокальциноз	CCAL2	АД	ANKH	Избыток ФРФ23	Мутации в генах стимулируют избыточную транскрипцию ФРФ23	#118600#123000
Краниометафизарная дисплазия, синдром Джексона	CMDJ
Остеосклеротическая костная дисплазия (Синдром Рейна)	RNS	АР	FAM20C	Избыток ФРФ23.	Мутантный белок Fam20c не способен фосфорилировать ФРФ23 для инактивации	#259775
Мутации рецепторов ФРФ23
Остеоглофоническая дисплазия		АД?	FGFR1	Резистентность к ФРФ23	Резистентность к ФРФ23.	#166250
Ненаследуемые заболевания
Опухоль-индуцированная остеомаляция	TIO		FN1-FGFR1, FN1-FGF1	Избыток ФРФ23	Гиперсекреция ФРФ23 опухолевыми клетками	
Хроническая почечная недостаточность	CKD			Избыток ФРФ23	Компенсаторное повышение ФРФ23	

**Figure:**
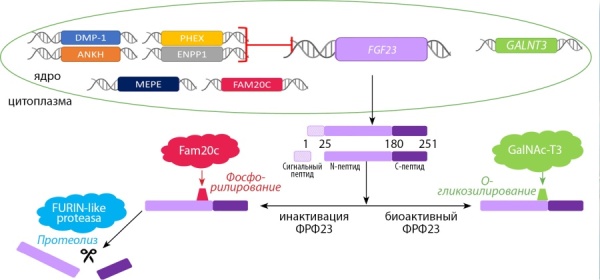
Рисунок 1. Синтез ФРФ23 в остеоците. Регуляция экспрессии ФРФ23 на уровнях транскрипции и посттрансляционных модификаций.Комментарий. Ген FGF23 расположен на 12-й хромосоме и кодирует белок ФРФ23 (251 амк) в остеоцитах. Процесс транскрипции ингибируют белки DMP-1, ANKH, PHEX, ENPP1. В процессе посттрансляционной модификации отщепляется сигнальный пептид (24 амк). Часть ФРФ23 инактивируется внутри клетки путем фосфорилирования белком Fam20C (FAM20C), а затем расщепляется белком FURIN-like proteasa на N-терминальный (154 амк) и С-терминальный (73 амк) пептиды, которые затем секретируются в кровь. Инактивации ФРФ23 препятствует процесс О-гликозилирования ФРФ23 белком GalNAc-N3 (GALNT3). О-гликозилированный ФРФ23 недоступен для протеолиза, тем самым повышается его концентрация крови. ФРФ23 — фактор роста фибробластов 23; GalNAc-T3 — полипептид N-ацетилгалактозаминилтрансфераза 3, GALNT3 — ген полипептида N-ацетилгалактозаминилтрансфераза 3; фосфор — неорганический фосфат.Figure 1. Synthesis of FGF23 in the osteocyte. Regulation of FGF23 expression at the levels of transcription and post-translational modifications.

После посттрансляционных модификаций в кровь секретируются нижеследующие формы ФРФ23. Во-первых, это биоактивный «интактный ФРФ23» (N+C-пептиды). Во-вторых, это 2 короткие, небиоактивные формы: С-пептид и N-пептид. Их роль ифункции изучаются. Современные лабораторные наборы в большинстве случаев определяют интактный ФРФ23. Однако есть тесты, которые оценивают С-пептид. Таким образом, измерение соотношений интактного и С-пептида ФРФ23 может помочь в диагностике ФРФ23-обусловленных нарушений (рис. 2) [43].

**Figure:**
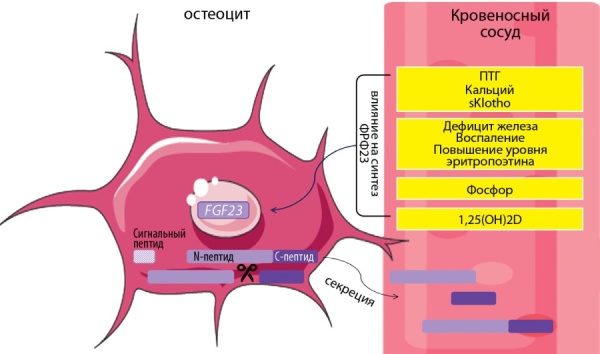
Рисунок 2. Секреция и регуляция концентрации ФРФ23 в системном кровотоке.Комментарии. После отщепления сигнального пептида (24 амк) секретируется интактный ФРФ23 (227 амк). Часть ФРФ23 инактивируется белком-протеазой (FURIN-like proteasa). В системный кровоток ФРФ23 секретируется в 3 формах: интактный (биоактивный) ФРФ23, С-пептид ФРФ23, N-пептид ФРФ23. 1,25(OH)2D, ПТГ, фосфор, кальций, sKlotho повышают концентрацию ФРФ23. Дефицит железа, воспаление могут параллельно увеличить как транскрипцию ФРФ23, так и его расщепление. ФРФ23 — фактор роста фибробластов 23.Figure 2. Secretion and regulation of FGF23 concentration in the systemic circulation

В крови также существует система контроля концентраций ФРФ23, однако процесс его инактивации до конца не ясен. Предполагают участие ингибиторов активатора плазминогена (plasminogen activator inhibitor-1 (PAI-1)). PAI-1 является ингибитором сериновой протеазы (SERPIN), известна его взаимосвязь с патологическими состояниями человека, включая хроническую болезнь почек, инфаркт миокарда и диабет [45].

Активно изучается влияние на концентрацию ФРФ23 системных факторов: 1,25(OH)2D (1,25 дигидроксихолекальциферол, кальцитриол), ПТГ (паратиреоидный гормон), фосфора, кальция, sKlotho (serum soluble Klotho, растворимый в сыворотке Клото), факторов воспаления, дефицита железа и эритропоэтина (рис. 2). 1,25(OH)2D, ПТГ, фосфор, кальций повышают концентрацию ФРФ23. Известно, что 1,25(OH)2D стимулирует транскрипцию ФРФ23 путем связывания в промоторной области генаFGF23 [43]. Остальные механизмы менее ясны. Кальций увеличивает транскрипцию ФРФ23, действуя через кальций-чувствительные каналы (L-type voltage-sensitive calcium channels) [46]. ПТГ опосредованно может способствовать транскрипции ФРФ23, действуя через циклическую AТФ-зависимую протеинкиназу (PKA), Wnt-NURR1 сигнальные пути [47][48]. Циркулирующие в крови sKlotho и ФРФ2 также стимулируют экспрессию ФРФ23, активируя рецепторы ФРФ на остеоцитах [49][50]. Дефицит железа может параллельно увеличить как транскрипцию ФРФ23, так и его расщепление [43]. Системное воспаление характеризуется низким содержанием железа в сыворотке крови несмотря на нормальные или повышенные запасы железа в организме, что приводит к аналогичному влиянию на ФРФ23 [43].

Биоактивная форма ФРФ23 разносится системным кровотоком и взаимодействует с рецепторами FGFR1-4, активируя RAS-MAPK и PI3K-AKT внутриклеточные сигнальные пути [31][51]. Способность связывания факторов роста фибробластов с тем или иным рецептором FGFR определяется наличием специфического ко-рецептора. Без наличия ко-рецептора аффинность ФРФ23 к рецепторам FGFR1-4 низкая. Основной функцией ФРФ23 является контроль уровня фосфора по аналогии с ПТГ, регулирующим уровень кальция. Фосфатурические эффекты ФРФ23 называются каноническими. Они осуществляются при взаимодействии ФРФ23 с рецептором FGFR1 подтипа С (FGFR1C) [13] и требуют обязательного наличия ко-рецептора, трансмембранного белка альфа-КЛОТО (KL, αKlotho) [52]. Органами-мишенями в таком случае являются почки и паращитовидные железы [52–54]. ФРФ23 регулирует баланс фосфатов, удаляя избыток фосфора с мочой, снижая активность натрий-зависимых фосфатных транспортеров NaPi2a и NaPi2c (SLC34a1 и SLC34a3), тем самым повышая экскрецию фосфора [21][55]. Кроме того, ФРФ23 ингибирует синтез 1,25(OH)2D в почках, снижая активность D-1α-гидроксилазы (CYP27B1), и усиливает его распад через активацию 24α-гидроксилазы (CYP24A1) [56]. В паращитовидных железах ФРФ23 ингибирует секрецию ПТГ, однако этот эффект зачастую преодолевается ФРФ23-опосредованной супрессией кальция, что влечет значительное повышение продукции ПТГ [57].

С течением времени были изучены новые, названные неканоническими, эффекты ФРФ23 на миокард, печень, иммунные клетки, костный мозг (рис. 3). Передача сигналов ФРФ23 в этих случаях происходит независимо от KL через рецепторы FGFR2-4 с участием других ко-рецепторов [58]. Активное изучение ФРФ23 продолжается на протяжении последних 20 лет, однако еще остаются нерешенные вопросы.

**Figure:**
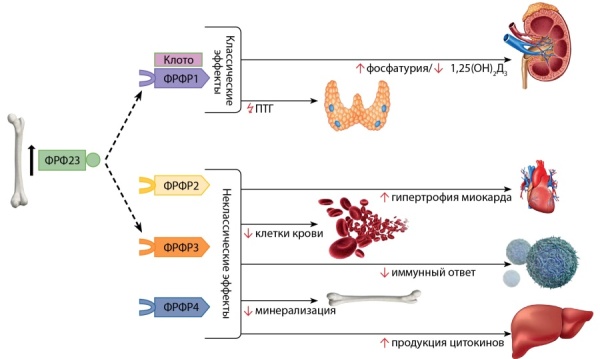
Рисунок 3. Эффекты ФРФ23.Комментарий. ФРФ23, взаимодействуя с рецептором 1c FGF (ФРФР1) и ко-рецептором альфа-КЛОТО (Kлото), регулирует баланс фосфатов, ингибирует синтез кальцитриола и опосредованно влияет на продукцию ПТГ. Изучаются новые эффекты ФРФ23 через взаимодействие с другими рецепторами (ФРФР2, -3, -4) и ко-факторами на миокард, печень, иммунные клетки, костный мозг.Figure 3. Effects of FGF23

При избытке ФРФ23 наблюдаются гипофосфатемия, дефицит 1,25(ОН)2D, что приводит к фосфопенической остеомаляции, зачастую с переломами скелета. Из-за дефицита 1,25(OH)2D может развиваться вторичный гиперпаратиреоз.

## ЭПИДЕМИОЛОГИЯ ОПУХОЛЬ-ИНДУЦИРОВАННОЙ ОСТЕОМАЛЯЦИИ

Опухолевой остеомаляции одинаково подвержены лица обоих полов, дебют заболевания наблюдается преимущественно в 40–45 лет, хотя встречается в любом возрасте [59–63]. Например, известны случаи пациентов с онкогенной остеомаляцией в возрасте 11,5 года [59], 3 лет [60][64] и даже 9 мес [62]. У детей преобладают генетические формы гиперсекреции ФРФ23 (PHEX-синдром, синдром Мак-Кьюна–Олбрайта, аутосомно-доминантный гипофосфатемический рахит и др., табл. 1) [15]. В случае выявления фосфопенической остеомаляции, индуцированной ФРФ23, необходимо исключать опухолевый генез. Настораживающими факторами в таком случае служат возраст и резкое начало заболевания.

Эпидемиология ФРФ23-опухолей остается малоизученной. По данным национального регистра Дании, с 2008 по 2018 гг. заболеваемость населения составила менее 0,13 на 100 000 человек, а у взрослых — 0,10 на 100 000 человек. Распространенность составила не более 0,70 на 100 000 человек среди всего населения и 0,43 на 100 000 среди взрослых. В 2018 г. среди взрослых было зарегистрировано не более 9 новых случаев. Код по МКБ-10 не определен, но чаще всего используются коды: М83.8 Другая остеомаляция у взрослых; E83.3 Нарушения обмена фосфора.

Клинические проявления онкогенной остеомаляции включают в себя значительные поражения опорно-двигательного аппарата c множественными переломами, выраженную мышечную слабость, которые в совокупности приводят к полной иммобилизации пациентов и развитию дыхательной недостаточности [1].

## ДИАГНОСТИКА ОПУХОЛЕВОЙ ОСТЕОМАЛЯЦИИ

## Лабораторная диагностика

В среднем от начала симптомов до постановки диагноза проходит 3 года, а до хирургического лечения — 5 лет [21]. За это время у пациентов успевают развиться переломы, деформации, выраженная хрупкость костей. В 95% случаев пациенты наблюдаются с диагнозами: остеопороз, гиперпаратиреоз, различные варианты артритов и другие заболевания минерального обмена, а также у неврологов из-за выраженной мышечной слабости [3]. Во избежание поздней диагностики этого редкого, но излечимого заболевания мы рекомендуем всем пациентам с остеопорозом, патологическими переломами и миопатией определять уровень фосфора в крови.

Характерными лабораторными признаками ФРФ23-опухоли в крови являются гипофосфатемия, повышение активности щелочной фосфатазы, нижне-нормальный уровень 25(ОН) витамина D и низкие концентрации активного метаболита 1,25(OH)2D, что может повлечь повышение уровней ПТГ и кальция [22][65][66]. При повышении уровня ПТГ необходима тщательная дифференциальная диагностика с гиперпаратиреозом, т.к. при длительном избытке ФРФ23 и реактивном повышении ПТГ развивается гиперплазия околощитовидных желез, которая ошибочно может трактоваться как первичный, а иногда и третичный гиперпаратиреоз. Далее оценивают потери фосфора с мочой. Для этого рассчитывают тубулярный индекс реабсорбции фосфора (TRP,%). В норме индекс реабсорбции фосфора должен составлять 85–95% [21]. При ФРФ23-опухоли фосфор активно экскретируется с мочой, и TRP,% составляет менее 85%. Расчет производится по представленной ниже формуле:

где TRP, % — тубулярный индекс реабсорбции фосфора;

UPh — фосфор разовой мочи (ммоль/л);

UCr — креатинин разовой мочи (ммоль/л);

PCr — креатинин плазмы либо сыворотки (ммоль/л);

PPh — фосфор плазмы либо сыворотки (ммоль/л).

* Если показатель креатинина представлен в единицах измерения мкмоль/л, то их следует перевести в ммоль/л. Для этого мкмоль/л необходимо разделить на 1000 и получить ммоль/л. Пример: креатинин 76,2 мкмоль/л = 0,0762 ммоль/л.

Пример:

дано: Фосфор разовой мочи — 11,72 (ммоль/л);

Креатинин разовой мочи — 3676,868 (мкмоль/л) = 3,676 ммоль/л;

Креатинин сыворотки — 76,1 (мкмоль/л) = 0,076 ммоль/л;

Фосфор сыворотки — 0,41 (ммоль/л).

При наличии хронической болезни почек стоит рассчитать индекс максимальной реабсорбции фосфатов с поправкой на скорость клубочковой фильтрации (СКФ) (TmP/GRF). Нормы приведены в таблице 2. Расчет TmP/GRF производится по однойиз двух формул, в зависимости от уровня TRP.

**Table:** Таблица 2. Референсные интервалы TmP/GRF ммоль/л (индекс максимальной реабсорбции фосфатов с поправкой на СКФ) [82]Table 2. TmP/GRF reference intervals mmol/L (maximum phosphate reabsorption index adjusted for GFR) [82]

Возраст	Мужской пол	Женский пол
Новорожденные	1,43–3,43	1,43–3,43
3 мес	1,48–3,30	1,48–3,30
6 мес	1,15–2,60	1,15–2,60
2–15 лет	1,15–2,44	1,15–2,44
25–35 лет	1,00–1,35	0,96–1,44
45–55 лет	0,90–1,35	0,88–1,42
65–75 лет	0,80–1,35	0,80–1,35

Если TRP≤0,86 (86%), тогда используют формулу:

TmP/GRF = PPh×TRP*,

где TmP/GRF — индекс максимальной реабсорбции фосфатов с поправкой на СКФ;

PPh — фосфор плазмы либо сыворотки (ммоль/л);

TRP — тубулярный индекс реабсорбции фосфора (в долях).

* Показатель TRP для расчета по формуле необходимо перевести в доли. Для этого TRP, % следует разделить на 100. Пример: TRP 41% = 0,41.

Пример:

Дано: Тубулярный индекс реабсорбции фосфора — 41% = 0,41;

Фосфор плазмы либо сыворотки — 0,41 (ммоль/л).

0,1681 = 0,41 х 0,41.

Если TRP≥0,86 (86%), тогда используют формулу:

где TmP/GRF — индекс максимальной реабсорбции фосфатов с поправкой на СКФ;

TRP — тубулярный индекс реабсорбции фосфора (в долях);

PPh — фосфор плазмы либо сыворотки (ммоль/л).

* Показатель TRP для расчета по формуле необходимо перевести в доли. Для этого TRP, % следует разделить на 100. Пример: TRP 41% = 0,41.

Пример:

дано: Тубулярный индекс реабсорбции фосфора — 87% = 0,87;

Фосфор плазмы либо сыворотки — 0,8 (ммоль/л)

После лабораторного подтверждения фосфопенической остеомаляции необходимо исключить наследуемые причины заболевания (табл. 1) [67], после чего можно приступать к топическому поиску новообразования. Наиболее известные варианты наследственных синдромов — мутации, влияющие на экспрессию гена ФРФ23 (врожденные рахиты с мутациями в генах FGF23, DMP-1, ENPP1, PHEX). Реже встречаются мутации рецепторов (FGFR1) и ко-факторов (Klotho), c которыми связывается ФРФ23. При невозможности провести генетическое тестирование необходимо ориентироваться на возраст пациентов, наследственность, характер заболевания [68][69].

## Инструментальная диагностика

Для успешного хирургического лечения ФРФ23-опухолей крайне важной является топическая диагностика. До 2017 г. в РФ в качестве метода диагностики применялись МСКТ либо МРТ всего тела, по результатам которых опухоли не всегда идентифицировались. В 2018 г. в России начали использовать более продвинутые методы диагностики, основанные на сродстве диагностических радиофармпрепаратов (РФП) к опухолевым рецепторам (соматостатиновые 2А типа SSTR2A) [1]. Применяется сцинтиграфия с РФП (99mTc-тектротид, 111In-октреотид) либо позитронно-эмиссионная томография (ПЭТ, ПЭТ/КТ) с РФП (соли галия: Ga DOTA-TATE). Широко используемый препарат для поиска новообразований 18F-фтордезоксиглюкоза (18F-ФДГ) не показал успешных результатов в диагностике ФРФ23-опухолей. Использование 18F-ФДГ обладает меньшей чувствительностью по сравнению со сцинтиграфией, а лучшим визуализирующим методом считается ПЭТ/КТ сGa DOTA-TATE [70]. Также отметим, что 21% опухолей не имеют на своей поверхности рецепторов SSTR2A, а значит, их поиск на данный момент затруднен. В мире описано применение селективного забора крови из вен с определением ФРФ23 в сомнительных (с точки зрения локализации опухоли) случаях [71][72].

Таким образом, инструментальная диагностика опухолей, секретирующих ФРФ23, строится поэтапно. Вначале проводят функциональную визуализацию с использованием специфических РФП. Затем осуществляют топический поиск опухоли: КТ, и/или МРТ, и/или УЗИ. При таком подходе удается диагностировать до 70% опухолей [72]. Образования обнаруживаются в мягких тканях (55%) или в костях (40%) любой локализации. Чаще поражаются конечности, голова и суставы. Размеры небольшие и составляют 1–2 см [73].

## Гистологическая диагностика и молекулярно-генетические особенности опухолей

Патоморфология ФРФ23-опухолей остается предметом активного изучения. Патоморфологическое исследование проводят после хирургического иссечения для подтверждения мезенхимального происхождения и оценки границ опухоли. Нe рекомендуется проводить предоперационную биопсию опухолей, т.к. при нарушении целостности образования возможно метастазирование по организму.

Гистологически фосфатурические мезенхимальные опухоли содержат остеокластоподобные гигантские клетки, множественные веретенообразные клетки, зрелый жир, гиперваскуляризованная капсула отсутствует, а опухоль может инфильтрировать кость между трабекулами и соседние ткани [16]. Злокачественные признаки (высокая клеточная и митотическая активность, некроз) не характерны.

Иммуногистохимически определяется экспрессия ФРФ23, рецептора соматостатина 2A (в 79%), FGFR1 (82%). J.-C. Lee и соавт. провели серию экспериментов и нашли типичные мутации в тканях опухоли — слияние генов FN1-FGFR1, реже FN1-FGF1(FN1 — фибронектин) [74]. Предполагается, что мутантный белок, продукт слияния генов FN1-FGFR1, обладает стимулирующим действием на FGFR1-рецепторы на поверхности опухоли и таким образом потенцирует рост и развитие образования.

## ТЕРАПИЯ ОПУХОЛЕВОЙ ОСТЕОМАЛЯЦИИ

Хирургическое лечение — это метод выбора при ФРФ23-опухолях. А первая операция — главный шанс излечить пациента. Резекцию опухоли необходимо по возможности проводить широко, в пределах здоровых тканей, особенно если образование не имеет капсулы и врастает в окружающие ткани. Единичные клетки, оставленные в послеоперационной ране, будут секретировать ФРФ23 и метастазировать. Рекомендуется избегать предоперационной биопсии и выскабливания опухоли по причине возможных метастазов. Желательно, чтобы операцию проводил опытный хирург, специализирующийся на пораженной области. Наиболее удобным показателем эффективности лечения является уровень фосфора, который восстанавливается уже на 5–10-е сутки после радикальной операции.

Отсутствие ремиссии может наблюдаться в следующих случаях:

Все вышеописанные причины являются показаниями к проведению консервативной терапии. Общепринятой на сегодняшний день схемой лечения пациентов с гипофосфатемической остеомаляцией является назначение альфакальцидола в дозах 4–4,5 мкг/сут, колекальциферола в поддерживающих дозах, по показаниям возможно применение препаратов фосфора 1–3 г/сут (суточную дозу распределяют на 3–4 приема в течение дня). Отметим, что препараты фосфора в России труднодоступны, а терапевтический ответ ограничен из-за побочных явлений, а именно диспептических расстройств (тошнота, рвота, диарея), развития нефрокальциноза и вторичного гиперпаратиреоза [75]. Многократный ежедневный прием (до 4–5 раз в сутки) солей фосфора снижает комплаентность пациентов.

Препарат буросумаб (KRN23), являющийся моноклональным антителом к ФРФ23, доказал эффективность и безопасность у данной группы пациентов (с 2020 г.), а также при врожденных рахитах с ФРФ23-опосредованным механизмом развития (с 2018 г.). Использование буросумаба продемонстрировало улучшение качества жизни, восстановление уровня фосфора, улучшение мышечной силы и гистоморфометрических параметров (снижение объема неминерализованного остеоида) [76]. Несмотря на то что буросумаб доказал свою краткосрочную эффективность, до сих пор отсутствует информация о его долгосрочных эффектах и о безопасности. Кроме того, поскольку буросумаб не останавливает прогрессирование или рост опухоли, его применение ограничено пациентами с неоперабельными или нелокализованными опухолями [2]. Буросумаб не зарегистрирован в России.

Идентификация транслокации FN1-FGFR1 в качестве молекулярного драйвера опухолей вызвала интерес научного сообщества [75]. Разрабатываются препараты с нацеливанием на FGFR1 для блокирования роста опухоли и секреции FGF23 [31][77–80]. Создан препарат ингибитор тирозинкиназы pan-FGFR BGJ398/инфигратиниб, который блокирует опухолевый FGFR1, что тормозит рост опухоли и секрецию ФРФ23. BGJ398/инфигратиниб уже показал свою эффективность, нормализуя ФРФ23 иснижая опухолевую нагрузку у пациентов с метастазами ФРФ23-опухоли [80]. Ингибитор pan-FGFR BGJ398/инфигратиниб является вариантом лечения, но из-за токсичности показан только пациентам с метастазами. Безопасность и эффективность применения ингибиторов FGFR являются областью активного изучения [31].

Цели консервативного лечения: в крови поддержание уровня фосфора в нижне-нормальных пределах, а показателей ПТГ, щелочной фосфатазы, кальция — в референсных интервалах. В качестве мониторинга исследуются показатели минерально-костного обмена каждые 3 мес. Кроме того, УЗИ почек проводится ежегодно, чтобы не пропустить развитие мочекаменной болезни на фоне медикаментозной терапии.

В литературе описаны альтернативные, но с недоказанной эффективностью методы лечения: препараты октреотида, цинакальцет, попытки радиочастотной абляции опухолевых очагов [81].

## ОБСУЖДЕНИЕ

Онкогенная остеомаляция является редким заболеванием, в связи с чем долго остается нераспознанной вплоть до наступления инвалидизирующих осложнений. Однако заподозрить диагноз можно, если измерить уровень фосфора в крови и рассчитать индекс реабсорбции фосфатов в моче. Чтобы не пропустить фосфопеническую остеомаляцию, вовремя отличить ее от остеопороза, мы рекомендуем измерять уровень как кальция, так и фосфора в крови всем пациентам спатологическими переломами и диагностированным остеопорозом.

Поэтапная инструментальная диагностика в большинстве случаев эффективна. Применение РФП, использующих экспрессию рецепторов соматостатина, отражает явный прогресс в визуализации, но все еще существует значительный процент опухолей, которые не удается обнаружить. Возможно, визуализация улучшится благодаря более специфичным лигандам к SSTR2A и усовершенствованиям детекторов. Также актуальной разработкой является поиск новых эпитопов на поверхности опухоли, таких как, например, мутантный белок, продукт слияния генов FN-FGFR1.

Развитие хирургических методов не стоит на месте, и опухоли, которые раньше были «неоперабельными», становятся доступны для резекции. Результаты хирургического лечения улучшаются благодаря распространению среди хирургов крайне важных фактов о том, что: 1) широкие резекции необходимы и приводят к стойкому излечению; 2) предоперационная биопсия опухолей не рекомендуется и может приводить к рецидивам; 3) опыт и профессионализм хирурга играют решающую роль в исходе операции. Кроме того, совершенствуются и исследуются менее инвазивные методы (радиочастотная абляция, лучевая терапия, селективный забор крови с определением ФРФ23 и радиохирургия). Если результаты испытаний подтвердят, что абляционные процедуры являются лечебными, то их можно будет использовать активнее [2].

На данный момент консервативная терапия улучшает качество жизни, состояние костной и мышечной массы благодаря возмещению дефицитов фосфора и витамина D. Очевидно, что возможности медикаментозного лечения улучшатся. Несмотря на то что буросумаб не влияет на рост опухоли, он, вероятно, станет эффективным средством для борьбы с заболеванием, с лучшей переносимостью и наименьшим количеством побочных эффектов. Ингибитор pan-FGFR BGJ398/инфигратиниб возможен как вариант лечения, но из-за токсичности его применение ограничено. В настоящее время разрабатывается ряд препаратов-ингибиторов FGFR1, pan-FGFR, которые могут быть эффективны при ФРФ23-опухолях. Однако, чтобы доказать свою эффективность и безопасность, они должны иметь ограниченную токсичность, что маловероятно [2][82].

## ЗАКЛЮЧЕНИЕ

Несмотря на огромные успехи, достигнутые в понимании патогенеза и терапии опухолевой остеомаляции, сохраняется необходимость в разработке более совершенных методов диагностики и лечения. Новые знания о молекулярных основах ФРФ23-опухолей вдохновляют на дальнейшие исследования. За последнее десятилетие в этой области достигнуты значительные успехи, и, видимо, их будет еще больше. Все это в совокупности дает надежду, что течение данного заболевания станет намного более благоприятным.

## ДОПОЛНИТЕЛЬНАЯ ИНФОРМАЦИЯ

Источники финансирования. Государственное задание № АААА-А20-120011690202-4 «Разработка персонализированных подходов к диагностике и лечению пациентов с остеопорозом вследствие эндокринопатий на основании изучения молекулярно-генетических предикторов, применения инновационных методов диагностики и исследования патогенеза редких заболеваний скелета».

Конфликт интересов. Авторы декларируют отсутствие явных и потенциальных конфликтов интересов, связанных с публикацией настоящей статьи.

Участие авторов. Все авторы внесли значимый вклад в проведение исследования и подготовку статьи, прочли и одобрили финальную версию статьи перед публикацией.
